# Supplementary material for: The role of eCIRP in bleomycin-induced pulmonary fibrosis in mice
Source: PLoS One. 2022 Apr 4;17(4):e0266163. doi: 10.1371/journal.pone.0266163 (PMC8979429; doi:10.1371/journal.pone.0266163)
Supplement: Attachment — Submitted filename: Respose to Reviewers.docx [file pone.0266163.s006.docx]

**Reviewer #1:** 
1) Clarify the established finding that no clear causal role is known for active inflammation in IPF.

Thank you for this suggestion. As recommended, we have included statements in the Introduction (line 54) and in the Discussion (lines 335-337) sections indicating that no clear causal role is known for active inflammation in IPF.

2) Remove discussion of Covid-19 unless it is directly relevant here.

The discussion related to Covid-19 has been deleted from the revised manuscript.

3) State that the assessment of fibrosis in mice was performed by persons blinded to the treatment/conditions. If not, then this assessment should be performed again in blinded fashion.

The histological assessment was performed in a blinded fashion. This information has been added to the Materials and Methods section (lines 153-154).

4) Make it very clear that studies in this mouse model cannot be used to make conclusions about human PF etiopathogenesis.

We have added a sentence clearly indicating that the bleomycin mouse model is not adequate for investigating the etiology of PF in humans (Discussion, lines 389-391).

Minor: In "Materials and Methods" The expression datasets were was obtained - please fix: The expression datasets were obtained.

The change has been made (line 76).

Bottom of page 6: Ten to twelve weeks-old male - please fix: Ten to twelve week-old male

The change has been made (line 89).

Page 11: that not only the expression of CIRP correlates - please fix: that not only does the expression of CIRP correlate

The change has been made. Thank you! (line 187).

**Reviewer #2:** 
1. There is a duplicate sentence in the abstract. Please edit.

Thank you for identifying this duplication! The change has been made.

2. Clarify the results section of the abstract, expression of CIRP increased during exacerbation of PF in human lung tissues. Unless you also tracked CIRP expression in mice over the course of bleo treatment and not just at day 22.

Thank you for this observation. The sentence was modified to “the expression of CIRP was higher in patients with acute exacerbation of PF than in patients with stable PF” (lines 38-39).

3. Clarify in methods what was harvested for qPCR, mouse lung and what lobes since portions were taken for histology and hydroxyproline assays?

The left lung was fixed in formalin for histological assessment and the right lung was used for qPCR (superior and middle lobes) and hydroxyproline content measurement (inferior and post-caval lobes). This information has been added to the Materials and Methods section (lines 102-104).

4. Did you notice any heterogeneity in the histological sections of mouse lung tissue? Could this variability account for the wide spread in your WT Bleo mice? Could you speak to why you might see such variability in these samples? Also was the scoring of the fibrotic lung tissue blinded?

The histological assessment was performed in a blinded fashion; this information has been added to the Materials and Methods section (lines 153-154). We did not observe histological heterogeneity that might explain the overall variability of the averaged Hübner-modified Ashcroft scores.

5. I would be curious to see if preemptive treatment or co-treatment with C23 with the bleo could further reduce or prevent the development of the fibrotic phenotype observed.

We would like to thank the reviewer for this insightful comment. At the design stage of this project, we did consider an arm of pre-treatment with C23. However, our group is more interested in new treatments for PF than in its etiology. As such, since pre-treatment is not a realistic therapeutic approach for PF patients, we decided not to proceed with this arm in the current project. Nonetheless, we will consider the Reviewer’s suggestion in our future studies.

6. I recommend including a section in the discussion about the application of C23 in patient treatment, potential issues, clinical use etc. to frame what is known and still needs to be understood.

Thank you very for that excellent recommendation. We have now included the sequence and source of C32 (Materials and Methods, line 106) and included the knowledge gaps that need to be addressed in reference to C23 (Discussion, lines 383-387).
